# The Effects of Noise on Human Cognitive Performance and Thermal Perception under Different Air Temperatures

**Published:** 2019-12-17

**Authors:** Shiva Sepehri, Mohsen Aliabadi, Rostam Golmohammadi, Mohammad Babamiri

**Affiliations:** ^1^Department of Occupational Health, School of Public Health, Hamadan University of Medical Sciences, Hamadan, Iran; ^2^Center of Excellence for Occupational Health, Occupational Health and Safety Research Center, Hamadan University of Medical Sciences, Hamadan, Iran; ^3^Center of Excellence for Occupational Health, Research Center for Health Sciences, Hamadan University of Medical Sciences, Hamadan, Iran .; ^4^Department of Ergonomics, School of Public Health and Research Center for Health Sciences, Hamadan University of Medical Sciences, Hamadan, Iran

**Keywords:** Psychomotor performance, Noise, Cold temperature, Thermal perception

## Abstract

**Background:** Environmental factors are interrelated, and human comfort is a complex state that is under the influence of all these factors perceived by a person. We aimed to investigate the effects of noise on human cognitive performance and thermal perception under different air temperatures.

**Study design:** An experimental study.

**Methods:** This study was conducted on 24 volunteers (12 males and 12 females) aged 18-30 yr old. All the experiments were carried out in a climate chamber located in Hamadan University of Medical Sciences in 2018. The subjects were exposed to ten different conditions set by a combination of three different air temperatures (14, 18, and 22 °C), three different noise levels (55, 65 and 75 dBA), and one irrelevant speech level in the climate chamber. The n-back, CPT, and PVT tests were employed to evaluate different aspects of cognitive performance. Thermal comfort and thermal sensation were measured with subjective questionnaires.

**Results:** With increasing noise under different air temperatures, working memory (*P*=0.001), sustained attention (*P*=0.001), and simple reaction time (*P*=0.001) were significantly disturbed. The combined effects of noise and low air temperature on working memory, sustained attention, and reaction time were higher than the effect of each of them individually. As compared with air temperature, noise has a larger effect on working memory, sustained attention, and reaction time in the test configurations.

**Conclusion:** The cognitive performance effects from noise has one veto power over these effects from low air temperature. Speech sound had more negative effects on subjects’ cognitive functions than the typical noise caused by office equipment. The subjective thermal perceptions were also influenced by noise at lower air temperatures.

## Introduction

There are several physical factors including noise, air temperature, lighting, etc., in indoor environment and it can affect the health, comfort, and performance of individuals. The concept of "health", according to the WHO, is "a state of complete physical, mental and social well-being and not merely the absence of a disease or infirmity" (https://www.who.int/about/who-we-are/constitution). On the basis of this broad definition, physical factors are important, because they can change the state of health by causing a lack of concentration, fatigue, decreased mental performance, dissatisfaction, and discomfort^1^. Noise and air temperature are the most important indoor environmental factors, which affect human health and mental performance^2^. For example, exposure to noise can have immediate and delayed effects. One of the main effects of exposure to noise is the decline in cognitive functions, which largely contributes to the occurrence of occupational accidents. Exposure to extreme cold and hot environmental temperature can also affect cognitive functions and lead to undesirable effects and health-related outcomes ^1^.

So far, some studies have investigated the effect of physical factors on cognitive function. Easterbrook stated that the exposure to noise can increase arousal so that it exceeds an optimum value and consequently, reduce the level of attention^3^. Among 58 studies conducted on the effects of noise on cognitive functions, 29 studies reported the negative effects of noise, 22 indicated that noise had no effect on cognitive functions, and 7 cases showed an improvement in cognitive performance^4^. Job performance and satisfaction were affected by physical and environmental factors in the work environment^5^. Moreover, noise had a negative effect on accuracy and reaction time^6^.

The effects of cold air on mental functions have also been studied using different cognitive tests. In general, simple tasks, as compared with complex ones, are more negatively affected by cold air exposure. Most of the effects documented to be related to cold temperatures have reported an increase in the number of errors and changes in response times in cognitive performance tests^7^. Exposure to cold (below 65 °F) had the most negative effect on performance in reasoning, learning, and memory tasks. The findings of meta-analysis have also shown a reduction of 7.81% in cognitive function in cold conditions (50- 64.9 °F) and a reduction of 13.91% in cognitive performance in colder conditions (less than 50 °F), as compared with neutral thermal conditions (65-75 °F) ^8^. Cold was associated with a decrease in cognitive performance. However, some studies have also reported evidence of improved performance in some cognitive tasks under moderate cold conditions ^9^.

In recent years, some studies have investigated the simultaneous exposure to noise and air temperature and its effect on human body responses in simulated indoor environments as same as office building. Noise significantly affected thermal comfort; moreover, thermal conditions also significantly affected noise sensations ^2^. Thermal comfort was affected by noise, while noise perceptions were not affected by ambient air temperatures ^10^.High levels of noise increased thermal discomfort ^11^. The increase in air temperature within a range of 22-30 °C and under a noise level of 55 dBA had negative effects on office work ^12^. The satisfaction levels of both air temperature and noise have one-vote veto power over the satisfaction level of the other indoor environmental factors such as lighting ^13^.Performance loss was observed, especially in working memory tasks, when the subjects were exposed to highly intelligible speech and high air temperature ^14^. Abbasi et al. observed the highest level of reduction in working memory when exposed to an air temperature of 30 °C and a noise level of 75 dBA ^15^.

The ASHRAE Guideline 10P highlighted the interactions between indoor environmental factors and the possible ways that various physical factors might affect each other; it also recommended to conduct more detailed researches ^16^.

Most studies have concentrated on the investigation of the effects of exposure to noise on cognitive performance in the context ofneutral air temperature or even high air temperature. We aimed to investigate the effects of noise on human cognitive performance including working memory sustained attention and simple reaction time under low air temperature conditions in a simulated indoor environment.

## Methods

### Participants 

This experimental study was conducted on 24 volunteers (12 males and 12 females) from among students in Hamadan University of Medical Sciences. Their mean (±SD) age, height, weight, and body mass index were 22.25 ± 2.38 yr, 169.87 ± 8.29 cm, 65.70 ± 8.64 kg, and 22.68 ± 1.76 kg / m^2^ respectively. To increase the accuracy of the study, the participants were screened using the self-reported questionnaire in terms of their state of mental and physical health including lack of a history of drug use and smoking, lack of a history of taking heart medications, antidepressant, sedatives, and other medications, lack of color blindness, having normal hearing and vision, not having a history of cardiovascular disease, respiratory problems, and sleep disorders, educational conditions, weight, height, and other demographic characteristics.

Before starting the tests, the study was approved by the Ethics Committee of Hamadan University of Medical Sciences (ethics code: IR.UMSHA.REC.1396.773), and a written informed consent form was signed by volunteers participating in this research.

All the subjects were paid in order to encourage them to perform the experiments seriously and correctly. A day before the test, the subjects were recommended to have enough sleep and rest, maintain a regular diet, and avoid taking medicines, coffee, and caffeine and they were also asked to turn their mobile phones off at the time of the tests. On the day of the test, a questionnaire collecting demographic data was distributed among the subjects and completed by them. The tests were performed using a within-subject design, so that all the subjects were tested in ten experimental conditions, thus acting as their own controls.

### Experimental setup

All the experiments were carried out in a climate chamber located in Hamadan University of Medical Sciences. The dimension of climate chamber was L×W×H=3.70×2.40×2.70 m and it had a workstation consisting of a desk, a chair, and a computer. To play the noise, two 10-watt speakers were used and one 8-watt subwoofer (Mingo MG-202) was placed on both sides of the monitor at a half-meter interval. It was possible to set and fix the chamber's thermal conditions using an air-conditioning system located outdoor of the chamber which was able to adjust temperature from -10 °C to 50 °C. The thermometer sensors installed on the room's wall continuously monitored the thermal conditions including air temperature and relative humidity inside the room during the experiment. When controlling the thermal conditions of the room, the room air temperature and relative humidity feedback were regularly obtained from these thermometer sensors. Thermal conditions are relatively homogenous in whole chamber for preventing subjects’ local thermal discomfort.

The chamber was equipped with two LED lights that provided fixed optimal lighting of about 300 lux for typical office building. The wall of the chamber was made of pre-made panels of injected polyurethane.

All the experiments were performed from Jun to Sep in 2018. Ten scenarios were designed. Nine scenarios included the exposure to combined physical parameters of fan noise at three different levels of 55, 65 and 75 dBA, and air temperatures at three different levels of 14, 18 and 22 °C; in addition, the tenth scenario included an irrelevant speech noise at a level of 75 dBA and at air temperature of 22 °C as relatively neutral thermal condition. Due to lack of the proper heating system and poor acoustic design, the selected noise levels and air temperatures scenarios can happen in typical office buildings.

Low-frequency noises originated from different sources such as fans are one of the most annoying and common complaints that, in addition to industrial environments, are also heard in environments such as administrative, commercial, and office environments ^6^.

In order to observe the ASHRAE recommendation published in 1992, which presents the optimum thermal comfort conditions in winter and summer ^17^, as the research was done in summer in this study, the thermal adaptation was set at 24 °C before the entrance of the subjects to the climate chamber. The volunteers were exposed to physical agents in the chamber for ten sessions (ten separate days) and each session lasted for 60 min. This experiment was carried out using a within-subject design. All the cognitive tests were taught to each person three times, and training exams were taken to ensure that they well understood the procedures. Each participant started the test at a pre-scheduled time of the day, and there was only one participant in the room each session. The possible carryover effects were partially controlled by random exposure to different scenarios. All the cognitive tests were performed using a personal computer. The level of lighting at the chamber was kept at a desirable level during all the tests. The relative humidity of the chamber was controlled and set at a fixed level of 50% and air velocity was set at a level less than 0.2 m/s. Prior to each test, the combined conditions of noise and temperature were set. In addition, a digital sound level meter (CASSELA CEL 450) was used to measure the noise level and a digital WBGT meter (CASSELA MTH-1) was also used to measure air temperature. In this study, the subjects remained seated and performed light work. In keeping with ISO 8996 standard, the metabolic rate for office work (light, sitting like typing) is 70 w/m^2^, equivalent to 1.2 met ^18^. In all the scenarios, consistent with ISO 9920 standard, the clothing insulation was set at 0.75 clo ^19^. The subjects entered the chamber and were exposed to the designed combined conditions for thirty minutes. During this period, books and magazines were at their disposal, after that they began to perform the tests. The first cognitive test was the n-back test (n=2) which lasted for five minutes. After that, the subjects had a rest for five minutes to prevent mental fatigue and then performed the continuous performance test for five minutes. At the end of the test, they rested for five minutes again and performed the psychomotor vigilance task for five minutes. Finally, thermal sensation and thermal comfort were measured with questionnaires for five minutes for each scenario.

### Cognitive performance measurement

The cognitive performance parameters employed in this research included working memory, sustained attention, and simple reaction time.

### N-back working memory task

To evaluate the performance of working memory, the visual n-back cognitive task, which is a software-based task, was used. The n-back task is an executive function measurement task that is commonly used in nerve imaging studies to stimulate the brain function of subjects. This task was first introduced by Kirchner in 1958. The overall trend of this task is to provide a sequence of stimuli (visual or auditory) to the subject step by step and the subject should check whether the current stimulus matches the stimulus presented in the preceding step. This test is performed using different values of n and with increasing n, the difficulty of the task increases as well. Thus, in the 1-back cognitive task, the last presented stimulus is compared with the previous stimulus and in the 2-back and 3-back, the last stimulus is compared with two and three previous stimuli, respectively.

Since in this task both maintaining cognitive information and their manipulation are evaluated, it is commonly used for measuring the performance of working memory and it has been extensively used in recent years. Its various types are well suited for experimental studies of working memory. For example, the validity of this test was very acceptable to measure the performance of the active memory ^20^.

The n-back test has three levels. In this study, only the 2-back task was used, because the 1-back task is a simple task not affected by changes; in addition, in the 3-back task, the changes may not be very different because of its complexity and difficulty. In this test (visual type), 120 numbers (from 1 to 9) were randomly displayed one after the other in the center of the computer screen for 5 min with a time interval of 1500 milliseconds. The participants were asked to compare each number with the two previous number if the numbers were similar, they were required to press the question mark button (?), and if different, they were asked to press the Z Button. The percentage of correct answers (accuracy) and the average response time (milliseconds) were recorded as dependent variables.

### CPT Cognitive Task (Continuous performance test) 

Continuous performance test is used to assess sustained attention. This test was introduced by Rosvold et al. and had quickly become popular ^21^. The purpose of this test is to measure the sustainability of attention and care. In all forms of the CPT test, the subject must pay attention to a relatively simple visual stimulus set for a while, and when he viewed the target stimulus, respond with the push of a key. In this test, 150 stimuli (visual type) were presented from which 20% were the target stimuli (the stimulus that the examinee had to give a response) on the computer screen. In this study, the target stimulus was the number 4. The time of presenting each stimulus was 200 milliseconds and the interval between each two presentations was 1 second. The number of correct answers, commission and omission errors, and the average response time (milliseconds) were recorded as dependent variables.

### PVT Cognitive Task (Psychomotor vigilance task)

In this study, the PVT task was used to measure the response speed. This task was developed during the Second World War to simulate radar surveillance operations. In this task, a red dot emerges in the middle of a computer screen, and subjects should respond to the stimuli quickly. In our study, this test consisted of red circles that appeared on the screen randomly with a certain time interval and the participants were asked to press the specified key (Space key) as soon as the target stimulus was presented. The stimuli were displayed in a black screen for 300 milliseconds and were presented randomly at time intervals of 2 to 10 sec. A software recorded the simple reaction time in milliseconds. PVT test is validated for measuring cognitive function, sleepiness, and fatigue ^22^.

### Thermal perception measurement

Based on the ISO 10551 standard, thermal sensation votes were cast on a 7-point thermal sensation scale as follows: cold (-3), cool (-2), slightly cool (-1), neutral (0), slightly warm (1), warm (2), and hot (3). Thermal comfort votes were also cast on a 4-point thermal comfort scale as follows: comfortable (0), slightly uncomfortable (1), uncomfortable (2), and very uncomfortable (3) ^23^.

### Statistical analysis

Data were analyzed using SPSS software (ver.22, Chicago, IL, USA). The normality of the data was tested using the Kolmogorov-Smirnov test. When data were normally distributed or when distributions were similarly skewed, they were analyzed using repeated-measures ANOVA or paired samples T-test. The Greenhouse-Geisser correction was applied when Mauchly’s test indicated the violation of sphericity, and the corresponding *P*-values were reported. Friedman and Wilcoxon’s tests were used when data were not normally distributed and were not similarly skewed. The significance level for all the results was set at 0.05 (*P*<0.05).

## Results

### Simple reaction time 

Table 1 presents the results of the PVT test for the different scenarios of exposure to noise and air temperature. Based on the results of ANOVA test with repeated measures, simple reaction time was significantly increased with increasing the noise levels at different air temperature (*P*=0.001). The effect size of increasing noise on simple reaction time at low air temperatures, as compared with that at neutral temperature, did not differ significantly. Moreover, with reducing the air temperature at the different noise levels, the simple reaction time was increased while the trend of changes was not statistically significant. The combined effects of noise and low air temperature on simple reaction time were higher than the effect of each of them individually. The effect sizes of increasing noise on simple reaction time at the different air temperatures were larger than the effect sizes of reducing the air temperature on the reaction time at the different noise levels (Table 1).

**Table 1:** The simple reaction time (ms) under different scenarios of exposure to noise and air temperature

**Noise levels**	**55dBA**		**65dBA**		**75dBA**		**Effect size**	**P value**
**Air temperature levels**	**Mean**	**SD**	**Mean**	**SD**	**Mean**	**SD**		
14 °C	329.20	30.45	337.91	30.03	347.37	30.60	0.610	0.001
18 °C	325.25	25.87	329.16	28.04	344.12	29.56	0.479	0.001
22 °C	321.91	21.53	333.33	24.63	341.62	25.85	0.571	0.001
Effect size	0.062		0.111		0.031			
*P* value	0.229		0.067		0.481			

### Working memory 

Tables 2 and 3 present the scores of the different aspects of cognitive performance obtained from the n-back test (percentage of correct responses and response time). In the n-back test at the different air temperatures, with increasing the noise levels, the percentage of the correct responses (accuracy) was reduced and the changes were statistically significant (*P*=0.001). Moreover, the results showed a significant difference between the correct responses at different air temperatures, so that with reducing the air temperature, the accuracy was reduced (*P*=0.001). With increasing the noise levels at different air temperatures, the response time was also increased, and the changes at 14 °C were not statistically significant (*P*=0.085), but at 22 °C, the changes in the response time were significant (*P*=0.002). As presented in Tables 2 and 3, generally, the effect sizes of increasing noise on accuracy and response time at the different air temperatures were larger than the effect sizes of reducing the air temperature on them at the different noise levels. The effect sizes of increasing noise on accuracy and response time at low air temperatures did not differ significantly from the effect sizes at neutral air temperature. The combined effects of noise and low air temperature on accuracy and response time were higher than the effect of each of them individually.

**Table 2:** The response accuracy (%) under different scenarios of exposure to noise and air temperature

**Noise levels**	**55dBA**		**65dBA**		**75dBA**		**Effect size**	**P value**
**Air temperature levels**	**Mean**	**SD**	**Mean**	**SD**	**Mean**	**SD**		
14 °C	88.87	3.41	86.33	3.77	82	6.62	0.547	0.001
18 °C	90.54	2.78	88.12	3.76	85.25	4.46	0.740	0.001
22 °C	91.58	2.55	89.12	2.84	87.37	3.53	0.685	0.001
Effect size	0.592		0.350		0.492			
*P* value	0.001		0.001		0.001			

**Note:** Accuracy: The percentage of correct answers

**Table 3:** The response time (ms) under different scenarios of exposure to noise and air temperature

**Noise levels**	**55dBA**		**65dBA**		**75dBA**		**Effect size**	***P*** **value**
**Air temperature levels**	**Mean**	**SD**	**Mean**	**SD**	**Mean**	**SD**		
14 °C	448.37	88.43	448.50	100.85	501.83	159.85	0.110	0.085
18 °C	422.16	77.21	447.83	83.44	458.29	81.09	0.182	0.010
22 °C	426.83	83.93	498.91	120.29	481.62	128.84	0.233	0.002
Effect size	0.088		0.131		0.052			
*P* value	0.120		0.053		0.286			

**Note:** Response time: The average response rate to the stimulus

### Sustained attention 

Tables 4, 5, 6 and 7 present the results of the CPT test under the different scenarios of exposure to noise and air temperature. The results showed significant differences between the number of correct responses, commission errors, and the response time at different noise levels so that with increasing the noise level, the mean number of correct responses decreased, and the mean response time and the number of commission errors increased. However, with increasing the noise level, there was no significant change in the number of omission errors at different air temperatures. The effect size of increasing noise on correct responses, commission and omission errors at an air temperature of 22 °C was higher than the effect size of increasing noise on the mentioned variables in an air temperature of 14 °C, and also the effect size of increasing noise on response time in an air temperature of 14 °C was higher than the effect size of increasing noise on the mentioned variable in the neutral temperature of 22 °C.

**Table 4:** The correct responses under different scenarios of exposure to noise and air temperature

**Noise levels**	**55dBA**		**65dBA**		**75dBA**		**Effect size**	***P *** **value**
**Air temperature levels**	**Mean**	**SD**	**Mean**	**SD**	**Mean**	**SD**		
14 °C	149.50	0.93	149.33	0.81	148.25	1.56	0.381	0.001
18 °C	149.66	0.56	149.16	0.63	148.62	1.13	0.362	0.001
22 °C	149.70	0.55	149.62	0.49	148.79	0.88	0.474	0.001
Effect size	0.031		0.129		0.079			
*P* value	0.454		0.042		0.150			

**Note:** Correct responses: The number of correct answers

**Table 5:** The response time (ms) under different scenarios of exposure to noise and air temperature

**Noise levels**	**55dBA**		**65dBA**		**75dBA**		**Effect size**	***P *** **value**
**Air temperature levels**	**Mean**	**SD**	**Mean**	**SD**	**Mean**	**SD**		
14 °C	425	33.79	432.83	37.26	446.45	38.60	0.457	0.001
18 °C	423.87	32.21	433.58	37.59	436.62	36.15	0.204	0.005
22 °C	411.95	33.58	421.41	28.91	425.58	30.76	0.230	0.006
Effect size	0.164		0.145		0.274			
*P* value	0.016		0.027		0.002			

**Note:** Response time: The average response rate to the stimulus

**Table 6:** The commission errors under different scenarios of exposure to noise and air temperature

**Noise levels**	**55dBA**		**65dBA**		**75dBA**		**Effect size**	***P*** **value**
**Air temperature levels**	**Mean**	**SD**	**Mean**	**SD**	**Mean**	**SD**		
14 °C	0.37	0.76	0.62	0.76	1.66	1.55	0.422	0.001
18 °C	0.29	0.55	0.79	0.58	1.29	1.12	0.353	0.001
22 °C	0.20	0.50	0.37	0.49	1.08	0.82	0.444	0.001
Effect size	0.031		0.127		0.094			
*P* value	0.584		0.060		0.073			

**Note:** Commission errors: The number of wrong answers

**Table 7:** The omission errors under different scenarios of exposure to noise and air temperature

**Noise levels**	**55dBA**		**65dBA**		**75dBA**		**Effect size**	***P*** **value**
**Air temperature levels**	**Mean**	**SD**	**Mean**	**SD**	**Mean**	**SD**		
**14 °C**	0.04	0.20	0.04	0.20	0.08	0.28	0.010	0.779
**18 °C**	0.04	0.20	0.04	0.20	0.08	0.28	0.010	0.779
**22 °C**	0.04	0.20	0.04	0.20	0.12	0.33	0.042	0.368
Effect size	0.001		0.001		0.010			
***P*** **value**	1.000		1.000		0.779			

**Note:** Omission errors: The number of missing answers

The results did not show a significant difference between the number of correct responses, commission and omission errors at different air temperatures when applying a background noise of 55 dBA and an annoying noise of 75 dBA; however, the differences between response times were significant. Generally, the effect sizes of increasing noise on the number of correct response, commission and omission errors and response time, at different temperatures were larger than the effect size of reducing air temperature on them. The combined effects of noise and low air temperature on correct responses, commission and omission errors and response time were higher than the effect of each of them individually.

### Effects of noise type 

In PVT test, the simple reaction time when exposed to irrelevant speech noise increased, as compared with the time exposing the subjects to the fan noise, and this change was statistically significant (*P*=0.049). In other words, this type of noise had more negative effects on the speed of responses than the typical noise caused by office equipment.

In n-back test, there were significant differences between the mean percentage of correct response (*P*=0.001) and the response time (*P*=0.004) when exposed to noise sources. The mean percentage of correct response, when exposed to irrelevant speech, has decreased as compared with the fan noise. Moreover, the mean response time when exposed to the fan noise was shorter than the mean response time when exposed to the irrelevant speech.

In CPT test, there were significant differences between the number of correct answers (*P*=0.001), commission errors (*P*=0.021), and response time (*P*=0.005) when exposed to noise sources. However, there was no significant difference between omission errors (*P*=0.317).

The average correct response, when exposed to irrelevant speech noise, decreased compared with the average correct response when exposed to fan noise. Moreover, the mean response time and the number of commission errors increased as well. Irrelevant speech noise was stronger than the fan noise in affecting cognitive variables of CPT test and the responses were worse.

### Thermal perception

The results of thermal sensation votes showed no significant differences between thermal sensation vote of the subjects with increasing noise at air temperatures of 18 (*P*=0.255) and 22 °C (*P*=0.981). However, based on different noise levels, the differences between thermal sensation vote were significant at 14 °C (*P*=0.018).

On the other hand, the results of thermal comfort votes showed no significant differences between thermal comfort vote of the subjects with increasing noise at air temperatures of 18 (*P*=0.071) and 22 °C (*P*=0.819). However, based on different noise levels, the differences between thermal comfort vote were significant at 14 °C (*P*=0.003). In other words, subjective thermal perceptions were influenced by noise at low air temperature.

## Discussion

Noise and air temperature are the most common causes of complaints in indoor environment so that the satisfaction levels of both air temperature and noise have one-vote veto power over the satisfaction level of the other indoor environment factors such as lighting ^13^. However, further studies are required to clarify the combined effects of them especially the simultaneous exposure to noise and low air temperature conditions.

In the present study, the findings of cognitive tests (n-back, PVT, CPT) showed that the cognitive functions significantly changed with increasing noise at air temperatures of 14 and 22 °C. The increase in noise had a destructive effect on cognitive performance. This finding is consistent with the results of some previous studies. Abbasi et al. studied the effects of low-frequency noise on cognitive performance and showed that the working memory reduced with increasing noise ^24^. High-frequency noise reduced memory performance more than the low-frequency noise ^25^. Memory and attention are heavily affected by noise, which in turn affects the ability of the subject to process information ^26^.

The safety and performance might be negatively affected when cognitive performance is impaired in working in cold conditions. According to our results, different aspects of cognitive performance were disturbed by reducing air temperature when the subjects were exposed to different noise levels. The effect of cold exposure was investigated on cognitive performance (accuracy, efficiency, and reaction time) and the exposure to cold had a negative impact on performance in both simple and complex tasks requiring concentration and sustained attention ^7^. Working memory, selective reaction time, and executive function decreased when exposing the subjects to a temperature of 10 °C, and these types of capabilities are interceded by frontal brain regions known to be negatively affected by cold stress^27^. Neurobehavioral tests were used to measure performance under the three temperature conditions of 17, 21 and 28 °C and the performance decreased when the thermal environment deviated from neutral conditions ^28^. Working memory performance was significantly affected by temperature conditions and the mean errors at 29°C were significantly higher than that at 21 and 25 °C^29^.

The evidence obtained from the present study showed that exposure to a combination of noise and temperature factors interactively affected cognitive performance (simple reaction time, working memory and sustained attention). With increasing the noise level and decreasing air temperature, the mean cognitive responses significantly changed and their destructive effect on cognitive performance increased.A mutual interaction was reported between noise and air temperature at harmful levels which increased the adverse effect of each of them ^30^. A negative interaction was found between the office noise and the moderate temperature, as the noise reduced the negative effects of the moderate temperature on the performance ^12^. However, the effects of noise and heat on the performance are independent ^31^. Taking into account the differences between the findings of various studies, it is reported that when different air environmental factors interact with each other, they reduce the performance. If their combined effect exceeds their individual effect, they exacerbate each other's effects and have the same mechanism of action. However, if the combined effects of risky environmental factors are equal with the individual effect of each of them on the performance, it is likely that the mechanism has a separate effect ^32^.

In this study, the effect of increasing noise on cognitive responses was larger than the effect of reducing the air temperature. This finding is consistent with other results, which reported the larger effect of noise on working memory, as compared with the air temperature in their study configuration ^15^.

The Yerkes-Dodson law is an empirical relationship between arousal and performance, originally developed by psychologists Robert M. The law dictates that performance increases with physiological or mental arousal, but only up to a point. When levels of arousal become too high, performance decreases ^33^. In this study, optimal cognitive performance was observed at an optimum temperature of 22 °C and a noise level of 55 dBA. The performance was reduced at lower air temperatures (14 and 18 °C) and the higher noise levels (65 and 75 dBA). Our findings confirmed that exposure to high noise level and low air temperature may increase arousal and lead to an impairment in the performance. Pilcher concluded that an air temperature of 26.67 °C and above at one end and an air temperature of 10 °C and lower at the other end (two vertices of the curve), when accompanied by an increase in motivation, resulted in an increase in the performance, indicated by the Inverted-U theory. However, if the motivation exceeds beyond a certain level, the performance will be degraded and will be less affected at a temperature range from 21.11 to 26.61 °C^8^.The optimum levels of air temperature (21 °C) and lighting (1000 lux) have improved the work performance ^34^. The optimum range of air temperature for performance was 22 – 26 °C^35^. The exposure to warm air temperatures and high levels of noise resulted in increased levels of arousal and decreased the cognitive function of individuals^15^.

The results showed that when exposing the subjects to irrelevant speech noise, as compared with fan noise, different aspects of cognitive performance disturbed. In other words, the performance of the subjects when exposed to fan noise was better than that when exposing the subjects to the irrelevant speech noise. Irrelevant speech is a type of noise shown to inﬂuence different elements of cognitive performance in a number of short-term memory tasks. The findings of this study are in line with the results of some previous studies, such as a study that found the highest reduction in performance when the sound source was intelligible speech (6.8%), followed by unintelligible speech (3.4%), printers (2.7 %), and ultimately ringing of telephones (1.9%) ^36^. Varjo et al. also observed a performance loss, particularly in work memory tasks, in subjects under simultaneous exposure to irrelevant speech, air temperature, and ventilation rate ^14^. Highly intelligible irrelevant speech disrupted the performance of working memory ^37^.

In this study, no significant changes were found in the evaluation of subjective thermal responses, with increasing noise levels from 55 dBA to 75 dBA at air temperatures of 18 and 22 °C, but the changes at 14 °C were statistically significant. Subjective thermal perceptions were influenced by increasing noise at lower air temperature. These results are consistent with some studies. Noise did not affect the cold sensations, but it affected thermal comfort and discomfort ^38^. Yang et al. also found the effects of noise on thermal sensation to be significant, although the effects of noise were minor ^39^.

The current study had some limitations. Based on our study design, short term exposures to different scenarios may create limited human responses ^40^. Hence, longer exposures times and repeated sessions of the different scenarios are encouraged. Moreover, field studies are needed to show cognitive performance in occupations that require long term exposures in adverse thermal environments. It is suggested future researches focus on the effects of the mentioned factors on other important aspects of cognitive performance. It is recommended to investigate the details of interactive effects of these stressors on human responses using advanced objective methods (e.g. *electroencephalography*). Finally, the results of this study could provide some evidence to help experts in designing and setting comfort standards for office buildings.

## Conclusion

With reducing air temperature and increasing noise, working memory sustained attention, and simple reaction time were disturbed. The results indicated interactions between the two main factors of noise and low air temperature at harmful levels that can increase the undesirable effects of each other. The combined effects of noise and low air temperature on working memory, sustained attention, and reaction time were higher than the effect of each of them individually. Generally, the effect size of increasing noise on cognitive variables was greater than the effect size of reducing air temperature on the mentioned variables in our study configuration. Taking into consideration the observed effect sizes, as compared with the relatively neutral air temperature of 22 °C, in an air temperature of 14 °C, the increase in noise did not aggravate cognitive performance. In general, the exposure to cold air did not largely enhanced the impact of noise on cognitive responses. As compared with the exposure to fan noise, the exposure to irrelevant speech had a more negative effect on cognitive responses. Optimal cognitive performance was observed at the relatively neutral temperature of 22 °C and a noise level of 55 dBA, as compared with other conditions. Perception of thermal environment was affected by noise changes at low air temperature. In other words, subjective thermal perceptions were influenced by noise at low air temperature.

## Acknowledgements

The authors would like to express their thanks to the participants who volunteered for this study.

## Conflict of interest

The authors declare that there are no conflicts of interest.

## Funding

This study was financially supported by Research Deputy of Hamadan University of Medical Sciences (Grant No. 9612087893).

## Highlights

- Optimal cognitive performance was observed at the relatively neutral temperature of 22 °C and a noise level of 55 dBA.
- The subjective thermal perception was affected by noise changes at low air temperature.
- Exposure to irrelevant speech had a more negative effect on cognitive responses.
- Noise has a greater effect on cognitive performance than air temperature.
